# Comparative Analysis of Lipid Extracts and Imaging Mass Spectrometry for Evaluating Cerebral White Matter Biochemical Pathology in an Experimental Second-Hand Cigarette Smoke Exposure Model

**DOI:** 10.4172/2469-9861.1000113

**Published:** 2016-04-20

**Authors:** Alexander Krotow, Emine B Yalcin, Jared Kay, Suzanne M de la Monte

**Affiliations:** 1 Department of Pathology, Brown University, Providence, RI, USA; 2 Liver Research Center, Department of Medicine, Rhode Island Hospital and the Alpert Medical School of Brown University, Providence, RI, USA; 3 Division of Neuropathology, and Departments of Pathology, Neurology, Neurosurgery and Medicine, Rhode Island Hospital and the Alpert Medical School of Brown University, Providence, RI, USA

**Keywords:** MALDI, Mass spectrometry, Tobacco, White matter, Neurodegeneration, Smoking, Mouse model

## Abstract

**Background:**

White matter injury and degeneration are common features of developmental and aging-associated diseases, yet their pathobiological bases are poorly understood. However, recent advances in Matrix-Assisted Laser Desorption Ionization (MALDI) instruments and chemistry have provided critical tools for myelin-lipid analytical research.

**Design:**

This study characterizes Cigarette Smoke (CS) exposure effects on frontal lobe lipid ion profiles in adult male A/J mice that had been exposed to air for 8 weeks (A8), CS for 4 (CS4) or 8 weeks (CS8), or CS8 followed by 2 weeks recovery (CS8+R). MALDI data acquired by analysis of lipid extracts plated onto a ground steel target (high through-put) were compared with Imaging Mass Spectrometry (IMS).

**Results:**

MALDI-time-of-flight (TOF) detected 120 lipid ions with m/z’s of 600 to 1300 (phospholipids and sulfatides) in samples plated onto the steel target or analyzed by IMS, but just 25 ions (18%) were detected by both methods. IMS more effectively detected ions in the highest m/z range, whereas the extracts had abundant middle-range m/z ions. The experimental groups were better discriminated by PCA and R-generated heat map hierarchical clustering of IMS data than lipid extract data. On the other hand, both methods clearly delineated the CS4, CS8 and CS8+R experimental groups from control.

**Conclusions:**

MALDI analysis of brain lipid extracts plated onto a ground steel target for high through-put studies, or imaged directly in tissue can be used to assess biochemical pathology of white matter neurodegeneration and responses to treatment.

## Introduction

Cerebral white matter degeneration is a major pathological feature of various neurodegenerative diseases including Alzheimer’s Disease (AD) [1–5], Parkinson’s dementia [5,6], fronto temporal lobar degeneration [7,8], alcoholic brain disease [9,10], and mitochondrial/metabolic diseases [11–13]. However, the pathogenic mechanisms of white matter degeneration remain poorly understood due to the lack of suitable tools to interrogate alterations in myelin and fiber tract integrity. Recently, we demonstrated that chronic Cigarette Smoke (CS) exposure causes white matter atrophy and degeneration with altered expression of myelin-associated genes [14,15]. Independent studies showed that smoking is a risk factor for cognitive impairment [16–18], brain atrophy [16,17,19–26], AD neurodegeneration [27,28], and white matter atrophy [18,26,29], and that white matter atrophy and degeneration develop early in the course of AD [5]. Since white matter myelin is largely composed of sphingolipids and phospholipids, characterizing brain white matter lipidomic profiles in relation to CS exposure duration and withdrawal could help distinguish different stages of disease and responses to treatment, and enrich our understanding of AD pathogenesis.

Rapid technological advances in Matrix-Assisted Laser Desorption Ionization (MALDI) have rendered it feasible to perform systematic lipidomics analysis of diseased brain white matter. However, an important consideration is the degree to which data generated with lipid extracts reflect in situ Imaging Mass Spectrometry (IMS) results. Initial high through-put analysis of tissue lipid extracts spotted onto a ground steel target could be used to screen samples for pathological effects and guide further selective IMS analysis of tissue sections. The present study compares the lipid ion profiles in extracts and fresh frozen histological sections of frontal lobe white matter in an experimental mouse model of second-hand CS exposure and withdrawal [30,31] in which significant CS-associated molecular and histopathological abnormalities have been demonstrated [14,15].

## Methods

### Experimental model

We utilized a second-hand Cigarette Smoke (CS) exposure model in male adult (8 weeks old) A/J mice (N=5–6/group). CS exposures were administered for 4 weeks (CS4), 8 weeks (CS8), or 8 weeks followed by 2 weeks recovery (CS8+R). Control mice were exposed to room air for 8 weeks (A8) [30,31]. To generate the model, Kentucky 3R4F research grade cigarettes (Tobacco Research Institute, University of Kentucky, Lexington, KY) containing 11 mg of total particulate matter (TPM) and 0.73 mg of nicotine were burned in Teague Enterprises, TE-10 Smoking Machine (Davis, CA). The CS exposures consisted of 89% side-stream and 11% mainstream smoke, mimicking environmental exposures. To accomplish this, 6 cigarettes were simultaneously puffed, once per minute and for 9 puffs, and cigarettes were burned for 6 hours/day, from Monday through Friday of each week. The peak CO levels was 24 ppm, which is comparable to that present in exhaled tobacco smoke (25–30 ppm) [32]. However, the CS exposure chambers had 21% oxygen and just 3 ppm of CO. All experiments were done according to University of Southern California’s Institutional Animal Care and Use Committee approved protocols which conformed to National Institutes of Health guidelines.

### MALDI plate assay

Lipids were extracted from frozen frontal lobe specimens (50 ± 5 mg) by the Folch method [33] after steel bead-based homogenization in sterile deionized water using a TissueLyser (Qiagen, Valencia, CA). After evaporating the organic phase to dryness in a SpeedVac vacuum centrifuge (Thermo Fisher Scientific, Waltham, MA), the pellets were dissolved in 100 μL HPLC grade methanol and stored at −80°C.

Saturated α-Cyano-4-hydroxycinnamic acid (HCCA, Sigma Aldrich, St. Louis, MO) prepared in TA50 (Acetonitrile/0.1% trifluoroacetic acid (TFA) in water, 50:50 v/v) was used as matrix and mixed 1:1 (v/v) with lipid extract. 1 μl aliquots were spotted into a 384-well ground steel MALDI Target Plate (MTP 384) (Bruker Daltonics, Bremen, Germany) and air-dried. The samples were analyzed in the negative ion mode of an Ultraflextreme MALDI-time-of-flight (TOF)/TOF (Bruker Daltonics, Bremen, Germany). Spectra data with the mass range set to 60–3500 Da were collected with rasterizing across each well and acquiring 150000 shots/well. Flex Analysis and tandem Mass Spectrometry (MS/MS) were performed on parent ions and lipid ion identifications were made based on assignments in the LIPID MAPS database (http://www.lipidmaps.org/tools/index.html). Statistical analysis was performed using ClinProTools, Version 3.0.

### MALDI-IMS studies

Frontal lobe cryo-sections (10 μm thick) were thaw-mounted onto Indium Tin Oxide (ITO)-coated slides (Delta Technologies, Loveland, CO) and prepared for lipid analysis by sublimation coating with 200 ± 13 mg/cm^2^ 2,5-dehydroxybenzoic acid (DHB; Sigma-Aldrich Co, St. Louis, MO) as a thin uniform layer of matrix [34,35]. Sublimation, a dry method that does not require a matrix solvent, is ideally suited for lipid analytes which could become delocalized. Matrix sublimation was achieved using a commercial apparatus (Chemglass Life Sciences, Vineland, NJ) in which 300 ± 5 mg of DHB crystals were evenly dispersed on the bottom of a lower flask that was tightly sealed to an upper flask that contained the MALDI target slide attached to its bottom surface with conductive copper tape. Under vacuum pressure (0.05 Torr) and with chilling of the upper flask, heat applied to the lower flask causes the DHB to transition from solid to gas phase, and subsequently condensate onto the surface of the cooled slide [35]. Imaging was performed with a reflectron geometry MALDI-TOF/TOF mass spectrometer (Ultraflextreme, Bruker Daltonics, Bremen, Germany). Analyses were performed by focusing a Smartbeam II Nd:YAG laser onto ~100 μm^2^ areas, with imaging data acquired in the negative ion mode [35,36].

### Data analysis

MALDI data were processed using Flex Analysis v3.4 (Bruker Daltonics, Billerica, MA) and visualized with Flex Imaging software v4.0 (Bruker Daltonics, Billerica, MA). Results were normalized to total ion count and analyzed statistically using ClinProTools v3.0 (Bruker Daltonics, Billerica, MA). Lipids were identified by comparing the precursor and product ion m/z values with those catalogued in the LIPID MAPS prediction tool database (http://www.lipidmaps.org/tools/index.html). Their identities were confirmed by tandem mass spectrometry (MS/MS) in the LIFT-TOF/TOF mode. Heatmaps were constructed using Version 3.2.2 of R software [37]. To scale the data, row means were subtracted from each cell. The resulting values were further divided by the standard deviation to obtain a z-score of each cell, yielding a mean of 0 and Standard Deviation (S.D.) of 1. The data were plotted using a cosmetically modified heat map library function in R with a 6-color palette. A hierarchical clustering algorithm using the Euclidean distance function was applied to the overall table to generate a lipid ions dendrogram.

## Results

### Lipid ion profiles detected in extracts and by IMS

A total of 120 distinct lipid ions with m/z of 600 to 1300 Da were detected by analysis of the extracts and/or by IMS; 83 ions were detected in extracts plated on the ground steel target and 62 ions were detected by IMS. Among those, 25 ions were detected by both methods (Table 1). In essence, 58 of the lipid ions detected in extracts were not detected by IMS, and 37 ions detected by IMS were not found in the extracts. TOF identified Phosphatidylserines (PS), Phosphatidylinositols (PI), and Sulfatides (ST) in data generated by both methods (Table 1).

**Figure 1:**
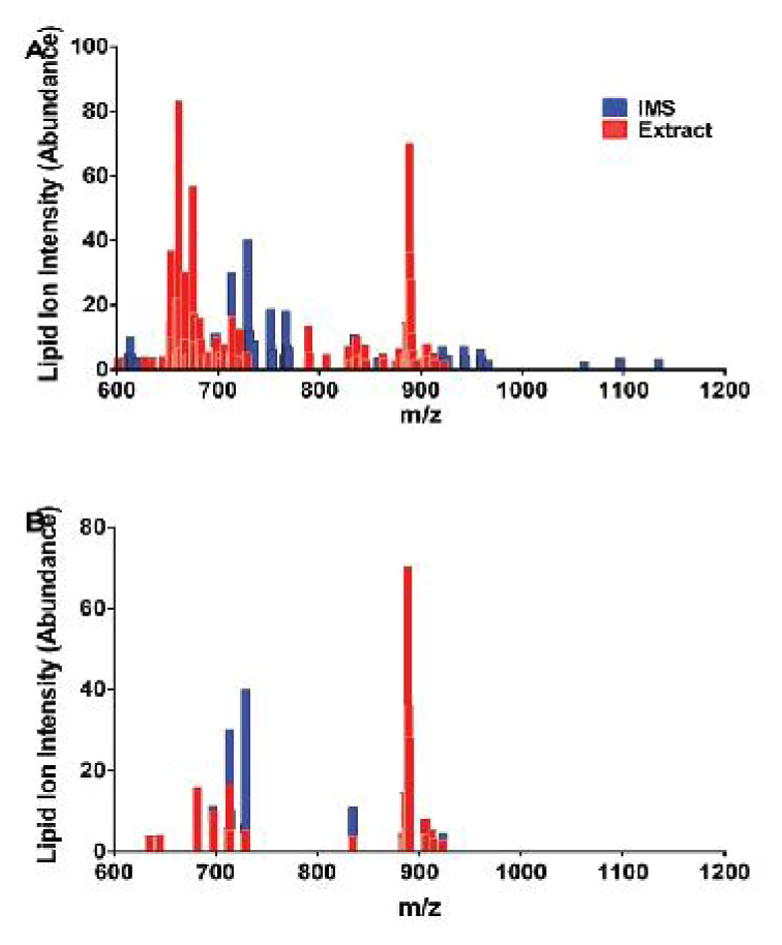
MALDI detection of lipid ion profiles in A/J control mouse frontal lobes. Fresh frozen histological sections and lipid extracts of frontal lobe tissue were analyzed by MALDI using Imaging Mass Spectrometry (IMS) or analysis of samples plated onto a ground steel target (extract). Lipid ion intensities (arbitrary units) were plotted for each m/z detected. Graphed data represent ion intensities (abundances) of the (A) full spectra for m/z’s from 600 to 1300 Da or (B) m/z’s corresponding to ions detected by both methods. Results reflect averaged data from 6 mice per group.

To better illustrate how the results differed according to the method of sample analysis, graphical comparisons of lipid ion m/z profiles and their relative intensities were made using data from the same A8 control samples analyzed as lipid extracts and by IMS (Figure 1). The superimposed plots (Figure 1A) revealed two clusters corresponding to the extracts: one with m/z’s between 627 and 729, and the other had m/z from 788 to 913. The IMS and extract profiles overlapped, but a number of higher m/z ions were detected in the IMS data. Lipid ions solidly within the mid-range of m/z’s were detected by both methods (Figure 1B), but the intensities/abundances of ions within the lower range cluster (m/z ~700) were higher for the IMS results, whereas within the higher range cluster (m/z 850–950) the intensities (abundances) were either comparable for the two approaches or higher in the extract data set. Among the lipid ions detected by both methods, 5 were similarly abundant (differences were less than 10%), 12 were more abundantly detected in the extracts, and 8 were more abundantly detected by IMS (Table 1).

### Comparative analysis of lipid ion peak profiles associated with CS exposures and withdrawal

**Figure 2:**
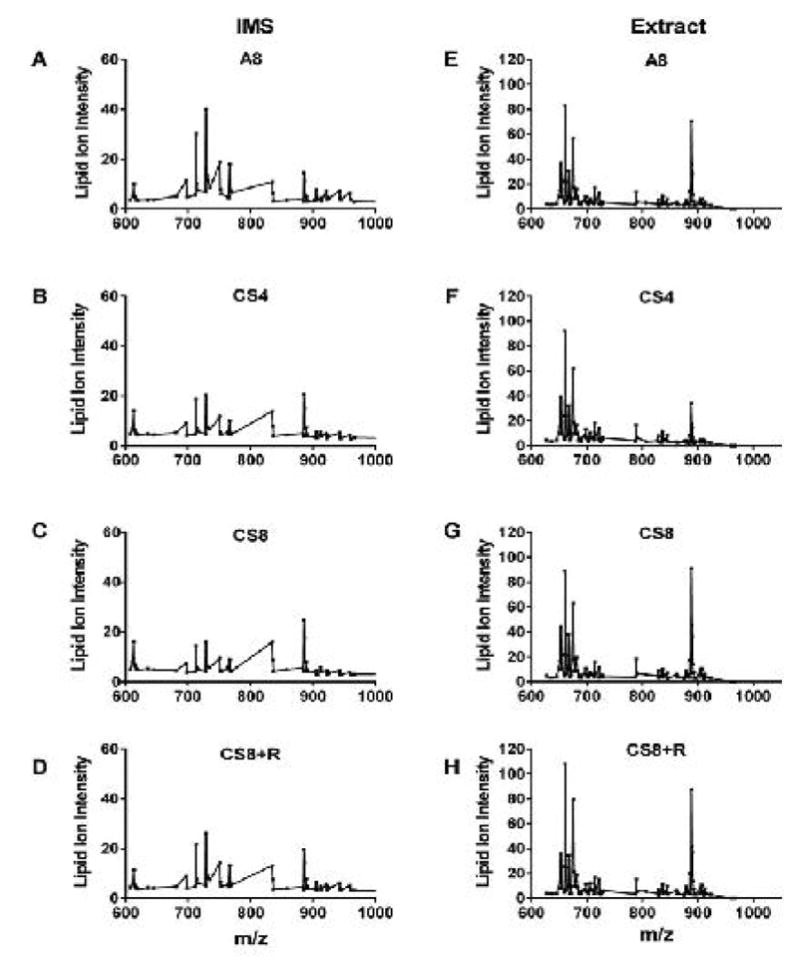
Effects of CS exposures and withdrawal on frontal lobe lipid ion expression as detected by MALDI IMS of fresh frozen histological sections (A–D) or in lipid extracts plated onto a steel target (E–H). Samples were obtained from A/J male mice exposed to air for 8 weeks (A8), cigarette smoke for 4 (CS4) or 8 (CS8) weeks, or 8 weeks followed by 2 weeks recovery (CS8+R). Lipid ion intensities (arbitrary units) were plotted for m/z’s between 600 to 1000 Da. Results reflect averaged data from 6 mice per group.

To illustrate the effects of CS exposure, duration, and withdrawal, IMS and extract data corresponding to lipid ion intensities for m/z’s from 600 to 1200 were plotted and aligned (Figure 2). The IMS profiles (Figure 2A–2D) were less complex than the extract profiles (Figure 2E–2H). Focusing on A8 control data, the IMS profile had greater abundances of lipid ions with m/z’s between 700 and 800, and above 900 (Figure 2A) compared with the corresponding extract data profile (Figure 2E). On the other hand, the control extract profiles exhibited complex clustering of ions with m/z’s between 650 and 700 and another around 800. CS exposures reduced the abundances of many lipid ions detected by IMS; exceptions included lipid ions with m/z’s of 600, 830, and 890, which increased with CS exposures (Figure 2B and 2C). CS8+R had somewhat of a normalizing effect in that it increased the lipid ions with m/z’s of 720, 740, and above 900 (Figure 2D), reducing the inhibitory effects observed in CS4 and CS8 samples. With regard to the extract data, the lipid ion peak profiles were strikingly similar across the 4 groups (Figure 2E–2H). One notable exception was that m/z 890 was reduced by CS4, increased by CS8, and normalized by CS8+R relative to A8.

### Principle component analysis (PCA)

**Figure 3:**
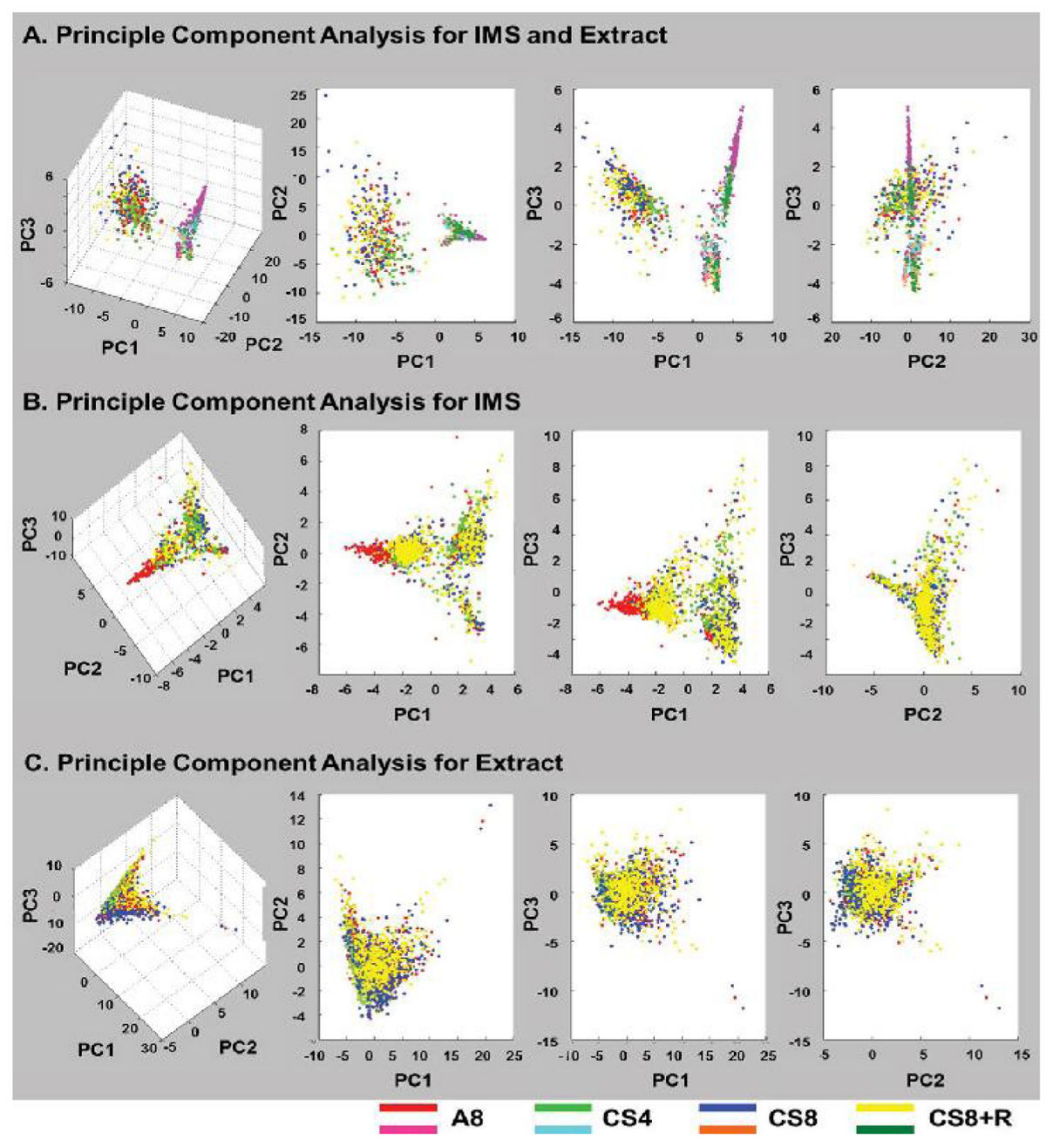
Principal component analysis (PCA) of extract and IMS data acquired in the negative-ionization mode. (A) 3-D (left) and 2-D (right 3 panels) plots showing separation of the extract and IMS results but tight clustering according to method of sample analysis. (B) IMS 3-D (left) and 2-D (right 3 panels) plots showing three distinct clusters for the A8 control (red), CS4 (green), and CS8 (blue) groups. CS8+R (yellow) overlaps with A8 as well as CS4 and/or CS8, reflecting partial reversal or blunting of CS effects. (C) Lipid extract 3-D (left) and 2-D (right 3 panels) plots illustrating only modest separation of A8 from the CS experimental groups.

Combined plots using data from both analytical methods revealed two distinct clusters, one corresponding to the extracts and the other to IMS (Figure 3A). Therefore, the two methodological approaches yielded distinct information. PCA of just the lipid extract data revealed extensive overlap with minimal separation of the A8, CS4, CS8 and CS8+R experimental groups on 3-D plots (Figure 3B). In contrast, the 2-D plot distinguished A8 controls from the CS groups, and although CS8+R overlapped with CS4 and CS8, its clustering was more compact and positioned over a single quadrant rather than dispersed like the CS4 and CS8 data (Figure 3C). In contrast, both the 3-D (Figure 3D) and 2-D (Figure 3E) PCA plots of the IMS data showed separation of A8 controls from CS4 and CS8, partial overlap of CS4 with CS8, and a clear shift of CS8+R toward A8, corresponding with trend reversal of CS’s effects after a brief period of withdrawal [14,15].

### Hierarchical clustering profiles

**Figure 4:**
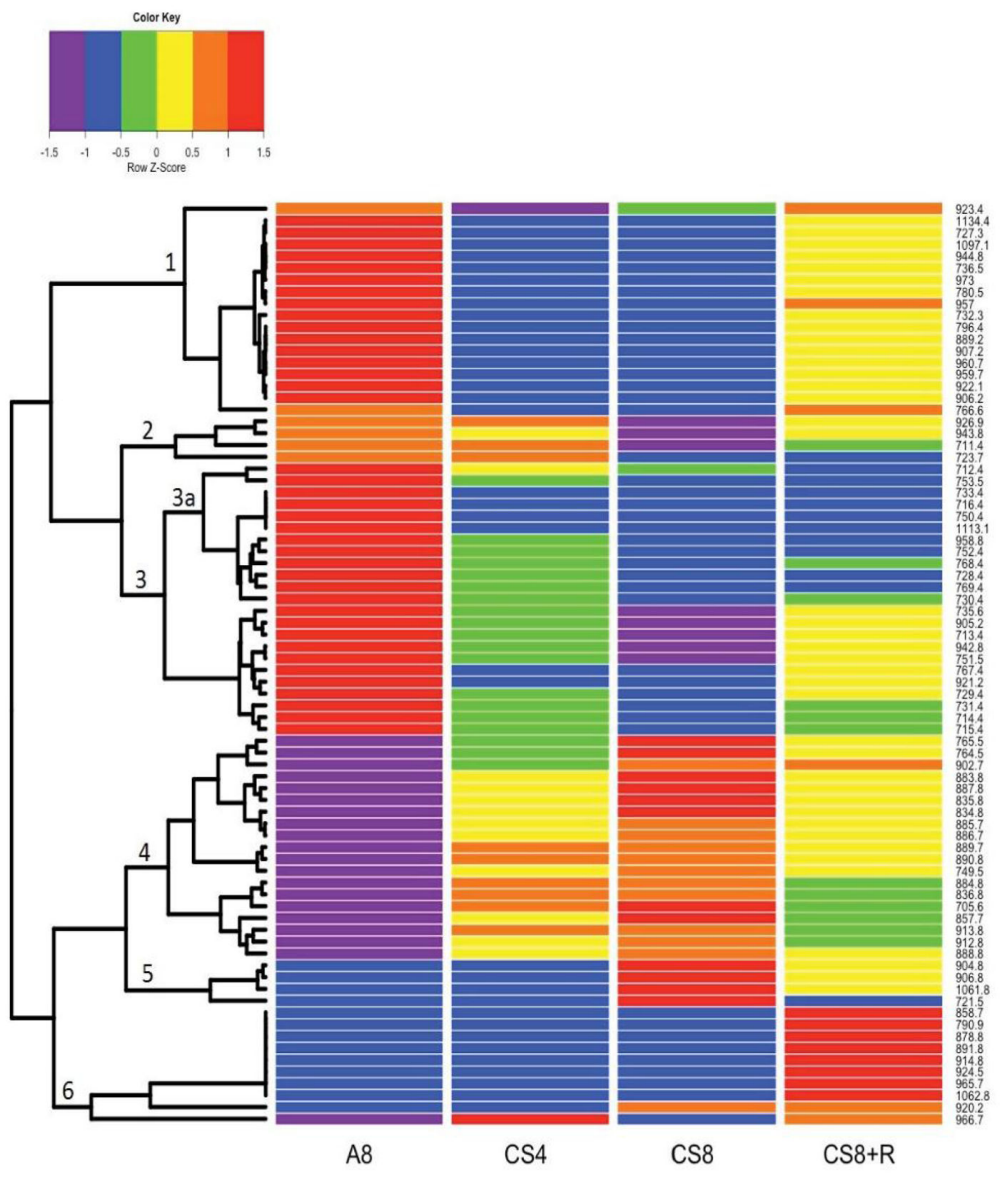

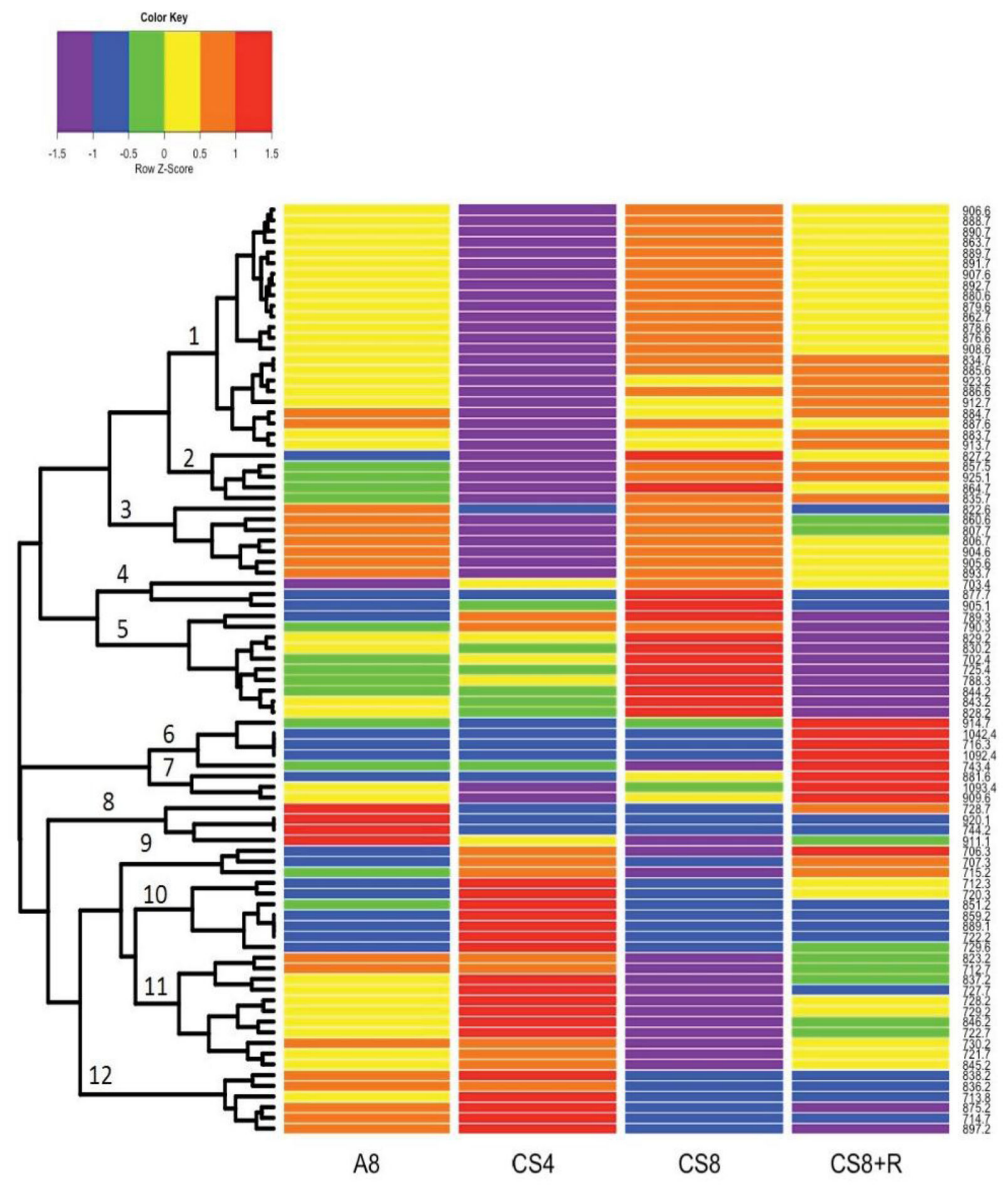
**a:** Heat maps of ions with m/z’s between 600 and 1300 Da detected by MALDI (A) IMS or (B) in lipid extracts of frontal lobe white matter from A/J male mice exposed to air for 8 weeks (A8), cigarette smoke for 4 (CS4) or 8 (CS8) weeks, or 8 weeks followed by 2 weeks recovery (CS8+R). Ions were clustered using a Euclidian-distance formula according to their behavior across experimental groups. Colors reflect z-value standard deviations (−1 to +1) from the mean (set=0) signal intensities (ion abundances) for each m/z value. **b:** Heat maps of ions with m/z’s between 600 and 1300 Da detected by MALDI (A) IMS or (B) in lipid extracts of frontal lobe white matter from A/J male mice exposed to air for 8 weeks (A8), cigarette smoke for 4 (CS4) or 8 (CS8) weeks, or 8 weeks followed by 2 weeks recovery (CS8+R). Ions were clustered using a Euclidian-distance formula according to their behavior across experimental groups. Colors reflect z-value standard deviations (−1 to +1) from the mean (set=0) signal intensities (ion abundances) for each m/z value.

R-generated heat maps with hierarchical dendrograms revealed relatively broad clustering of profiles by IMS (6 major groups) (Figure 4A) and more complex clustering with the extract data (12 main groups) (Figure 4B), corresponding to the lipid peak ion profiles shown in Figure 4. With regard to the IMS data, the upper three clusters are associated with very high lipid ion levels in the control group, sharply reduced expression in the CS4 and CS8 groups, and partial reversal or normalization of the levels (occurred in 60% of the lipids (Figure 4A). In Cluster 1, lipid ion expression was similarly low in CS4 and CS8. In Cluster 2, there was a progressive decline in lipid ion expression from CS4 to CS8 and minimal reversal in CS8+R. In Cluster 3, there was a progressive decline in lipid ion abundance from CS4 to CS8, but partial trend reversal after cessation of the CS exposures. The bottom 3 clusters showed very low levels of lipid ion expression in controls, and three types of responses to progressive increases (Cluster 4), similar degree increases (Cluster 5), or no increase (Cluster 6) in lipid ion abundance with duration of CS exposure. CS8+R’s therapeutic effects were manifested by declines in lipid ion abundance (Clusters 4 and 5) or sharply increased levels above the other 3 groups (Cluster 6).

Since the heat map corresponding to the extracts was extremely complex, only the main findings are described. In Clusters 1–4, intermediate to moderately high lipid ion levels were detected in A8, and the sharply reduced levels were measured in CS4 samples. However, the CS4 inhibitory effects were reversed by CS8 and CS8+R, resulting in higher or similar lipid ion levels relative to A8 (Figure 4B). In Clusters 5–7, lipid ion levels were similar in A8 and CS4, but differentially modulated by CS8 and CS8+R such that with regard to Cluster 5, CS8 sharply increased while CS8+R inhibited lipid expression, and with respect to Clusters 6 and 7, CS8 either had no effect or moderately increased lipid ion levels, while CS8+R sharply increased lipid ion expression. For Clusters 9–12, lipid ion expression was intermediate or moderately high in A8 control samples, highest or nearly highest in CS4 samples, and lowest in the CS8 group. The effects of CS8+R varied from nil relative to CS8 (Clusters 10 and 12), normalizing relative to A8 (Cluster 11), or similar to CS4 (Cluster 9). In essence, the 4 uppermost clusters showed inhibitory effects of CS4, stimulatory effects of CS8 and either normalizing or stimulatory effects of CS8+R. The 4 middle clusters showed minimal effect of CS4, striking stimulation of CS8, and inhibition by CS8+R. The 4 lowest clusters showed striking stimulatory effects of CS4, inhibitory effects of CS8, and either persistent inhibition or normalization trends for CS8+R.

### Comparative effectiveness of MALDI detection lipid ions in extracts and by IMS

**Figure 5:**
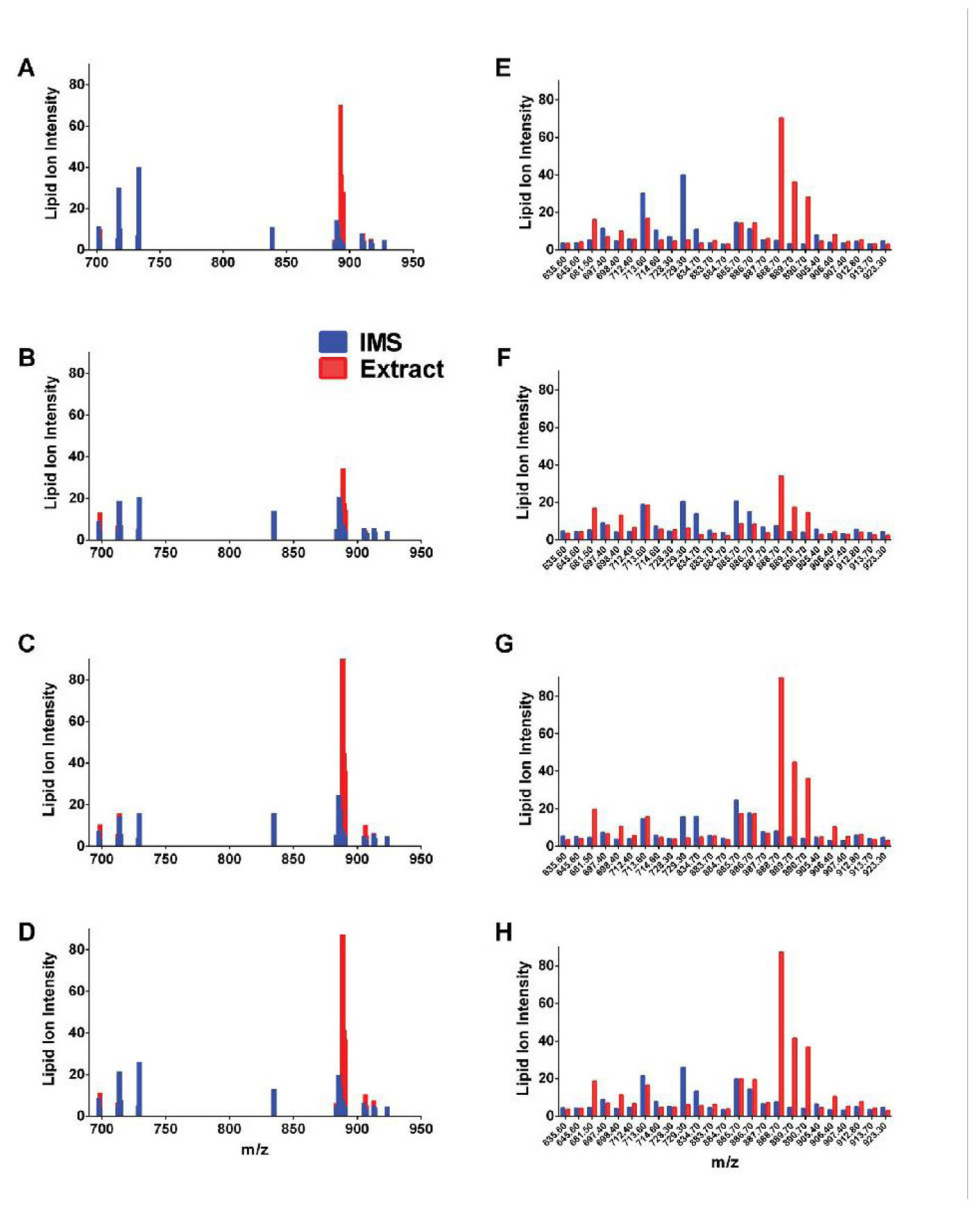
MALDI analysis of CS exposure and withdrawal effects on frontal lobe lipid ions detected by both IMS and in lipid extracts. A/J male mice were exposed to (A,E) air for 8 weeks (A8), cigarette smoke for (B,F) 4 (CS4) or (C,G) 8 (CS8) weeks, or (D,H) cigarette smoke for 8 weeks followed by 2 weeks recovery (CS8+R) (n=6/group). Samples were analyzed by MALDI using IMS of tissue sections or extracts plated onto a ground steel target. Signal intensities (arbitrary units) corresponding to m/z’s (600–1000 Da) detected by both methods are graphed with results (A–D) superimposed to scale according to m/z, or (E–H) illustrated side-by-side.

Since only a subset of lipid ions that had the same m/z were detected by MALDI analysis of the extracts and IMS, we further parsed the data to assess the strengths of each approach for detecting effects of CS exposure and withdrawal. The shared lipid ion peak intensities were graphed to either superimpose (Figures 5A–5D), or display side-byside (Figures 5E–5H) results. From the overlapped profiles, it is evident that IMS detected 3 ions with m/z’s 697.35, 713.75, and 729.22 (Figures 5A and 5E) that were sharply reduced by CS exposures (Figures 5B, 5C, 5F, 5G) and whose CS inhibitory effects were partly abrogated by CS withdrawal (Figures 5D and 5H), and another lipid ion at m/z 834.67 that was not appreciably modulated by CS exposure or withdrawal. The two sulfatide ions with m/z’s of 888.65 or 890.66, were abundantly detected in the extracts and were modulated by CS exposures such that both ions were suppressed by CS4 and either up-regulated or normalized by CS8 and CS8+R. Similar responses occurred with respect to 885.62 and 886.64, but their abundances were much lower than the ions with m/z’s of 888.65, 890.66. Therefore, IMS assessments of m/z’s 697.35, 713.75, and 729.22 report effects of CS exposures on the brain, and may also mark recovery following cessation of CS exposure. At the same time, the unchanging levels of m/z 834.67 in the IMS profile could provide an internal negative control or standard for future assessments. Finally, MALDI probing of brain lipid extracts registered inhibitory effects of CS4 on m/z’s 888.65, 890.66, 885.62, and 886.64 but not the long-term effects CS exposure or withdrawal. Note that the specific lipid ions modulated with CS exposure and withdrawal included phosphatidylserines, phosphatidylinositols, and sulfatides (Table 1).

## Discussion

This study demonstrates that CS-induced alterations in brain lipid ion profiles are detectable by MALDI via analysis of lipid extracts in high through-put assays or IMS. Although all lipid subtypes were detected using either approach, the results differed with respect to methodology. Both the number of distinct lipid ions and the profile complexity were greater in the extract than IMS data. Mid-range m/z ions (700–800) were readily detected by both methods, but ions with the highest or lowest m/z were better detected by IMS. In light of the excessive complexity of mid-range and virtual absence of high-range m/z ions in the extract profiles vis-a-vis considerably fewer ions and distinct cluster of high m/z ions detected by IMS, one consideration is that the tissue processing and/or lipid extraction procedures may lead to ion cleavage/fragmentation with artificially increased numbers of unique species. Furthermore, disease states such as neurodegeneration, which lead to endogenously increased oxidative stress in tissue could also contribute to increased macromolecular fragility, and artificially skew results. Fortunately, these problems will be circumventable as emerging methods such as Sequential Window Acquisition of all Theoretical Mass Spectra (SWATH) acquisition and TOF identification of parent plus all related fragment ions [38–41] become more widely available for diagnostics. Importantly, this MS/MS all approach can be applied to metabolomics including lipids [42]. An important goal of this type of research is to employ high through-put screening assays of tissue lipid extracts for subsequent focused IMS analysis of lipid ion profile alterations in relation to disease.

Another potential explanation for obtaining broader peak clusters by IMS compared with extracts is that minor (low abundance) lipid ion signals within the clusters may have been suppressed by neighboring dominant ions. However, this potential limitation is probably out-weighed by the advantages of in situ lipidomics in relation to histopathology. For example, brain white matter pathology often has a gradient such that the most prominent injury or degeneration occurs in periventricular zones rather than in central or subcortical regions. These effects could be readily demonstrated using MALDI-IMS, but would be lost by high through-put analysis of total lipid extracts unless the extracts were prepared from micro-dissected specimens, which would be highly tedious and labor-intensive.

The comparative analyses of lipid ion profiles demonstrated clear effects of CS exposure, duration, and withdrawal in data acquired by IMS, whereas the responses were less clear using steel target plated extract data. Similarly, PCA was more informative with respect to the IMS compared with steel target plated extract data results. These observations suggest that IMS may be more sensitive and reliable for distinguishing effects of different exposures and disease states on brain biochemistry. The explanation for this could be that the lipid ions which were “preferentially” detected by IMS are more prone to be altered in disease states. This is particularly relevant to the 800+ and below 700 m/z clusters (Figure 4). However, with regard to lipid ion detected by both methods, m/z’s 697.35, 712.3 and 728.2 were considerably more abundant in the steel target plated extracts whereas m/z 888.65 was the dominant ion detected by IMS (Figure 1). Since all four m/z were modulated by CS exposure and withdrawal, and were detected in both steel target plated extracts and by IMS, they represent potentially relevant biomarkers of myelin biochemical pathology in smoking-induced neurodegeneration. This suggests that MALDI analysis of samples spotted onto a steel target or subjected to IMS could be used to detect specific alterations in lipid ion intensities that correlate with disease or responses to treatment.

The broad effects of CS can be summarized as: 1) generally inhibitory or stimulatory irrespective of exposure duration; 2) progressively inhibitory or stimulatory with increasing exposure duration; or 3) transiently inhibitory or stimulatory after short-term CS exposure. CS withdrawal (CS8+R) had several effects ranging from reversal of CS-induced trends to paradoxically increasing or decreasing lipid ion expression. The hierarchical dendrograms revealed that variable size clusters of lipid ions exhibited similarly patterned responses to CS exposures, indicating that the effects were non-random and were likely mechanistically driven. The finding that the patterned responses within each cluster were distinguished from one another, further suggests that factors regulating expression of different lipid ions were differentially modulated by the same exposures. As noted with respect to the lipid ion profile plots and PCA, the IMS heat map more clearly delineated the effects of CS and withdrawal than did the steel target-extract data heat map, which was exceedingly complex due to the greater abundance of mid-range m/z lipid ions.

This study represents the first attempt to assess effects of CS exposures and withdrawal on white matter lipid ion profiles in the brain, comparing data from MALDI relatively high through-put and IMS assays. Among the 25 lipid ions detected by both methods, 8 proved to be informative of CS’s effects and 1 stood apart as relatively stable and unaffected by CS exposure. The exposure-related informative lipid ions were identified by MS/MS as phospholipids (phosphatidylserines or phosphatidylinositols) and sulfatides. Of interest is that the “diagnostic” ions that were more abundantly present in the extracts included both sulfatides and phospholipids, whereas those detected by IMS were exclusively phospholipids. Therefore, the diagnostic information obtained by each approach should be regarded as complementary rather than identical. The findings herein are exciting because they suggest that the use of MALDI for delineating white matter biochemical pathology can be applied to a broad range of central nervous system diseases. The next steps should be to characterize patterned alterations in white matter lipid ions that correlate with specific exposures, diseases, disease stages, and responses to treatment, and assess the degree to which the findings are disease-specific or general indices of neurodegeneration.

## Figures and Tables

**Table 1:** Lipid ions (600–1200 Da) detected by MALDI in lipid extracts and by imaging mass spectrometry.

m/z (Da)	Lipid Identification	Relative Abundance
635.6	*	IMS = Extract
645.6	*	IMS < Extract
681.5	*	IMS ≪ Extract
697.4	Phospholipid	IMS > Extract
698.4	*	IMS ≪ Extract
712.4	Phospholipid	IMS = Extract
713.6	Phospholipid	IMS > Extract
714.6	**	IMS ≫ Extract
728.3	Phospholipid	IMS > Extract
729.3	Phospholipid	IMS ≫ Extract
834.7	PS(40:6)	IMS ≫ Extract
883.7	PI(38:5)	IMS < Extract
884.7	**	IMS = Extract
885.7	PI(38:4)	IMS = Extract
886.7	PI(38:3)	IMS < Extract
887.7	Phospholipid	IMS < Extract
888.7	ST(24:1)	IMS ≪ Extract
889.7	**	IMS ≪ Extract
890.7	ST(24:0)	IMS ≪ Extract
905.4	**	IMS > Extract
906.4	ST(24:0)(OH)	IMS ≪ Extract
907.4	Phospholipid	IMS < Extract
912.8	PI(40:4)	IMS < Extract
913.7	**	IMS = Extract
923.3	*	IMS > Extract

Tandem MS with MALDI-LIFT-TOF/TOF was used to fragment lipids in the negative ion mode. Product ion spectra were identified using the LIPID MAPS database. PS=phosphatidylserine, PI=phosphatidylinositol; ST=sulfatide. Several phospholipid ions could not be further characterized due to their low abundances. In some instances (*), lipid identity could not be assigned due to unavailability of MS/MS data. In addition, lipid ions with m/z’s of 714.6, 884.7, 889.7, 905.4, and 913.7 (**) most likely correspond to C13 isotopes of the adjacent 1 Da-lower m/z ion. Relative Abundance: (=) IMS and Extract ion intensities differed by less than 10%; (> or <) IMS signal intensities were 10–49% higher or lower than observed in corresponding extracts; (≫ or ≪) IMS signal intensities were at least 50% higher or lower than in corresponding extracts. Lipid ion m/z’s highlighted in bold-italics have potential diagnostic utility in this model.
